# Backbone and side chain resonance assignment of the intrinsically disordered human DBNDD1 protein

**DOI:** 10.1007/s12104-022-10086-3

**Published:** 2022-04-26

**Authors:** Christoph Wiedemann, Kingsley Benjamin Obika, Sandra Liebscher, Jan Jirschitzka, Oliver Ohlenschläger, Frank Bordusa

**Affiliations:** 1 Charles Tanford Protein Centre, Institute of Biochemistry and Biotechnology, Martin Luther University Halle-Wittenberg, Kurt-Mothes-Str. 3a, 06120 Halle, Germany; 2 Department of Chemistry, Institute of Biochemistry, University of Cologne, Zülpicher Str. 47, 50674 Cologne, Germany; 3 Leibniz Institute on Aging - Fritz Lipmann Institute, Beutenbergstr. 11, 07745 Jena, Germany; 4 Faculty of Chemistry and Earth Sciences, Institute of Organic Chemistry and Macromolecular Chemistry, Biostructural Interactions, Friedrich Schiller University Jena, Humboldtstraße 10, 07743 Jena, Germany

**Keywords:** Dysbindin domain-containing protein 1 (DBNDD1), Dystrobrevin-binding protein, Intrinsically disordered protein (IDP), Solution state nuclear magnetic resonance, Solution NMR, Backbone and side chain nuclear magnetic resonance assignments, Chemical shifts

## Abstract

The dysbindin domain-containing protein 1 (DBNDD1) is a conserved protein among higher eukaryotes whose structure and function are poorly investigated so far. Here, we present the backbone and side chain nuclear magnetic resonance assignments for the human DBNDD1 protein. Our chemical-shift based secondary structure analysis reveals the human DBNDD1 as an intrinsically disordered protein.

## Biological context

The dysbindin (dystrobrevin-binding protein) protein family is a group of evolutionarily related proteins of moderate size (Mw 13–45 kDa) in higher Eukaryotes. Their amino acid sequences suggest that they are mainly cytosolic or nuclear proteins partly associating with membranes (Talbot et al. 2009). The human dysbindin protein sub-family consists of the supposed paralogs dysbindin-1 (alternative short names: DTNBP1, BLOC-1 Subunit 8 or HPS7 protein; UniProtKB: Q96EV8), dysbindin-2 (alternative short name: DBNDD2, CK1BP or HSMNP1; UniProtKB: Q9BQY9), and dysbindin-3 (alternative short name: DBNDD1; UniProtKB: Q9H9R9), with each of them expressing various isoforms. In humans this results in at least eight family members (dysbindin-1A, -1B, -1C, -2A, -2B, -2C, -3A, and -3B) currently reported (Talbot et al. 2009). The designated dysbindin paralogs show very limited sequence homology which raised the question whether DBNDD1 and DBNDD2 are dysbindin-like proteins or proteins that share a less conserved domain with DTNBP1 in the context of otherwise unrelated sequences (Ghiani and Dell’Angelica 2011).

Human dysbindin domain-containing protein 1 (DBNDD1) is encoded by the gene *DBNDD1* at chromosome locus 16q24.3. Currently, three human isoforms produced by alternative splicing are known for DBNDD1 (Bateman et al. 2021). The human DBNDD1 isoforms 1 (UniProtKB: Q9H9R9-1) and 2 (UniProtKB: Q9H9R9-2) differ only in the N-terminal region where 20 amino acids are additional in isoform 2. In contrast, isoform 3 (UniProtKB: Q9H9R9-3) carries a 100 amino acids long N-terminal sequence extension.

The canonical human protein DBNDD1 (UniProtKB: Q9H9R9), the focus of our study, is 158 amino acids long with a high content of the acidic residues glutamate and aspartate (13% and 7%, respectively) as well as serine (6%) and threonine (8%). Unlike other dysbindin family proteins, DBNDD1 isoforms are probably non-classical secretory proteins (Talbot et al. 2009).

Additionally, it is a proline-rich (10% prolines) cytoplasmatic protein with expression in nearly all organs and e.g., neuronal cells. No expression could be detected in the ovary, the adipose tissue, and the bone marrow [(Uhlen et al. 2015), https://www.proteinatlas.org].

The Pfam database [(Mistry et al. 2021), https://pfam.xfam.org/] predicts human DBNDD1 mainly as an intrinsically disordered protein (IDP) and also the recently released AlphaFold database (Jumper et al. 2021; Varadi et al. 2022) predicts human DBNDD1 – with a short stretch of helical propensity between residues L77 and S95 – entirely as an IDP. Interestingly, S95 (beside S119) is one of the two reported phosphorylation sites. Along with S65, S95 is proposed to constitute a casein kinase 1 interaction site while S119 might be modified by cyclin-dependent kinase 5 (Talbot et al. 2009).

**Fig. 1:**
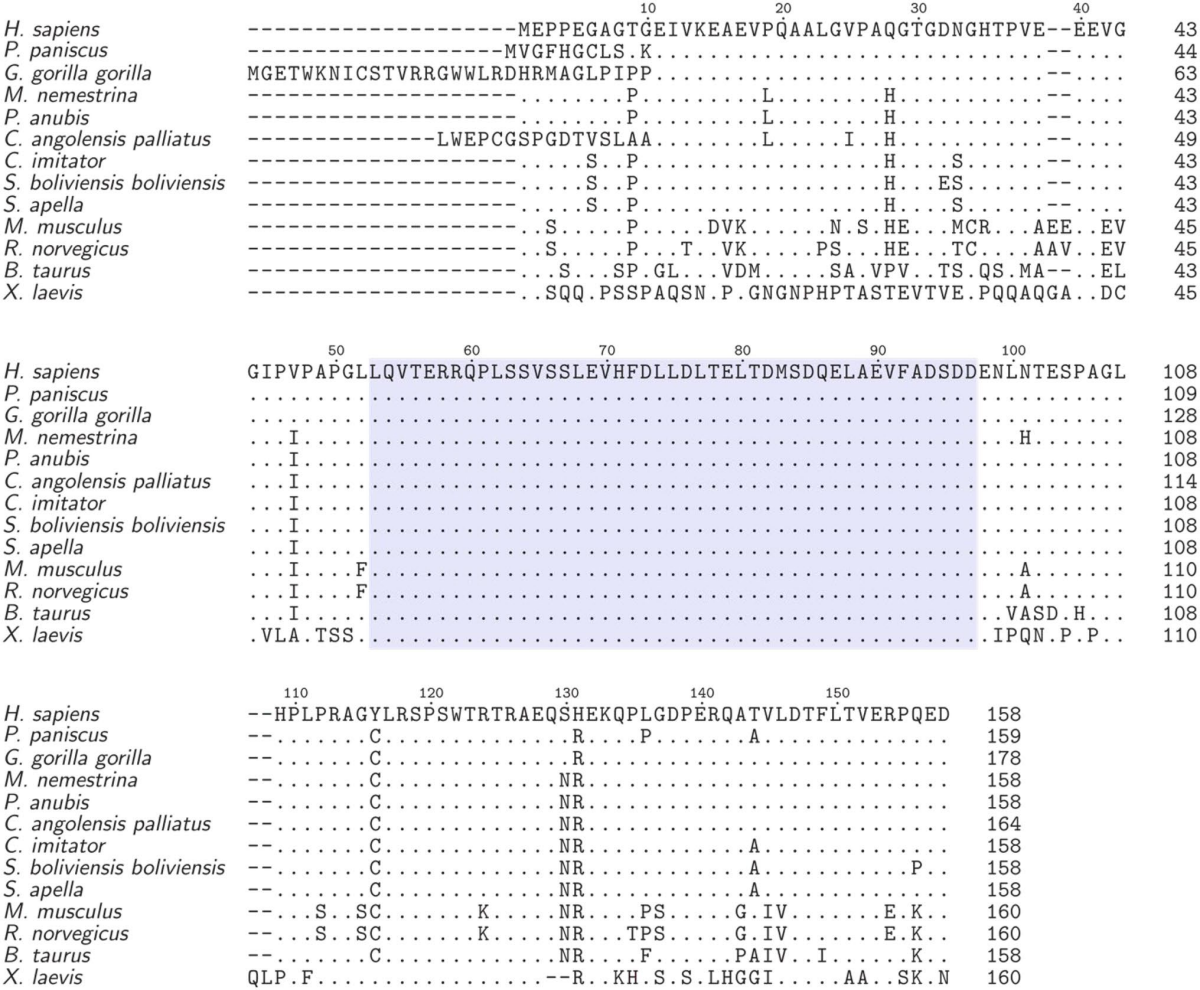
Dysbindin domain-containing protein 1 (DBNDD1) is conserved in different species. Sequence alignment [Clustal Omega (Madeira et al. 2019) was used, https://www.ebi.ac.uk/Tools/msa/clustalo/] of human DBNDD1 and similar protein sequences found by a BLAST search in other selected species. The human DBNDD1 sequence is used as consensus. Mismatches are shown in capital letters. Dashes indicate missing residues and dots represent identical residues in other sequences, respectively. The predicted dysbindin domain is highlighted. *H. sapiens* (Human, UniProtKB: Q9H9R9), *P. paniscus* (Bonobo, UniProtKB: A0A2R9AE53) and *G. gorilla gorilla* (Western lowland gorilla, UniProtKB: A0A2I2Y6M9) are Hominidae. Old World monkeys are *M. nemestrina* (Southern pig-tailed macaque, UniProtKB: A0A2K6BVD7), *P. anubis* (Olive baboon, UniProtKB: A0A096NR46) and *C. angolensis palliatus* (Angola colobus, UniProtKB: A0A2K5I6Q9). *C. imitator* (Panamanian white-faced capuchin, UniProtKB: A0A2K5QFU2), *S. boliviensis boliviensis* (Black-capped squirrel monkey, UniProtKB: A0A2K6U736) and *S. apella* (Tufted capuchin, UniProtKB: A0A6J3IDV6) represent selected New World monkeys. Additionally, DBNDD1-like proteins are also found in other species [e.g., *M. musculus* (Mouse, UniProtKB: Q9CZ00), *R. novegicus* (Rat, UniProtKB: Q5M831), *B. taurus* (Bovine, UniProtKB: A6H7B4) and, *X laevis* (African clawed frog, UniProtKB: Q6DJE5)]

We performed a Basic Local Alignment Search Tool (BLAST) analysis to identify regions of local similarity between the human DBNDD1 and protein sequences from other species (Fig. 1). As an outcome human DBNDD1 revealed a high sequence identity to dysbindin domain-containing proteins from other Hominidae (e.g., *G. gorilla gorilla* and *P. paniscus* 99% and 97% identity, respectively). Likewise, the proteome of Old and New World monkeys contains DBNDD1-like proteins with sequence identities to human DBNDD1 of approximately 95%. Proteins with high sequence identity to human DBNDD1 can also be found in evolutionarily more distant species (e.g., *M. musculus* and *X. laevis* 80% and 61% identity, respectively). The sequence conservation of the putative dysbindin domain across all selected species is notable (Fig. 1 shaded region).

Although, a high sequence conservation also suggests a conservation of structure and function, current experimental insights into the structure or function are missing on human DBNDD1 with the exception of some experimental data indicating that the *DBNDD1* gene is associated with melanoma risk and that the DBNDD1 level is decreased in Parkinson’s disease mouse models (Auburger et al. 2016; Fang et al. 2020). Also, a negative regulation of protein kinase activity is predicted for DBNDD1.

In contrast, its paralog dysbindin-1, the first family member discovered (Benson et al. 2001), is more intensively described. Dysbindin-1 contains a coiled-coil domain, a structural component known to e.g., facilitate biological maintenance, repair, replication, trafficking processes and enzymatic activities (Truebestein and Leonard 2016). Expression of dysbindin-1 is ubiquitous in the body and in virtually all neuronal cells (Talbot et al. 2009). For instance, dysbindin-1 was shown to be involved in neurite extension and synaptic vesicle trafficking (Auburger et al. 2016). Mutations in dysbindin-1 are responsible for the Hermansky-Pudlak syndrome (Li et al. 2003) and genetic variations of dysbindin-1 are associated with psychiatric conditions like psychosis, bipolar disorder, major depression, and schizophrenia (Straub et al. 2002; Talbot et al. 2009).

From the data available for DBNDD1 and its paralogs, it becomes clear that DBNDD1 may be involved in essential cellular processes. Thus, investigation of human DBNDD1 can broaden our understanding of the exact function of this protein and help to explain the previously observed associations with pathological manifestations.

## Methods and experiments

### Protein expression and purification

We ordered a synthetic gene coding for full-length human *DBNDD1* from Thermo Fischer Scientific (Germany). The coding sequence was optimized for expression in *E. coli*.

The gene was subcloned into a pET28a expression vector using Ndel and Xhol restriction enzymes, thereby introducing a N-terminal hexahistidine fusion. The resulting construct was verified by DNA sequencing (LGC Genomics GmbH, 120 Germany). For expression, transformed *Escherichia coli* BL21 (DE3) cells were plated onto kanamycin plates. A single colony was picked to inoculate a first LB-medium preculture. At an OD_600_ of 0.6 cells were diluted 1:50 in M9 mineral salts medium grown again. This step was repeated with fresh M9 medium. Subsequently, cells were diluted 1:70 in 250 mL M9 medium main culture supplemented with 1 g/l ^15^NH_4_Cl and 4 g/l ^13^C_6_-labeled glucose. Gene expression was induced at an OD_600_ of 0.6–0.8 by adding 1 mM IPTG (isopropyl-1-β-d-galactopyranoside). Cells were harvested after 4 h by centrifugation (5250xg, 30 min, 4 °C). All cultures were grown at 37 °C and supplemented with 50 μg/ml kanamycin.

For purification, cells were resuspended in 40 mL lysis buffer (11.5 mM Na_2_HPO_4_, 8.5 mM NaH_2_PO_4_, 500 mM NaCl, 10 mM imidazole, pH 7.0) containing a protease inhibitor cocktail (cOmplete Mini from Roche Diagnostics GmbH, Mannheim, Germany). Cells were disrupted by sonification while placed on ice and then centrifuged (40,000 rpm, 40 min, 4 °C, Beckman Coulter Optima L-90 K Ultracentrifuge). The supernatant was loaded onto a pre-equilibrated Ni–NTA affinity chromatography column (ÄKTA prime plus, QIAGEN Ni–NTA Superflow Cartridge 1 × 5 ml) at 4 °C. After washing with 10 column volumes of the lysis buffer human DBNDD1 was eluted with 11.5 mM Na_2_HPO_4_, 8.5 mM NaH_2_PO_4_, 500 mM NaCl, 500 mM imidazole, pH 7.0. Further purification was done by size exclusion chromatography (HiLoad 16/60 SD75, GE Healthcare) using 10 mM sodium phosphate buffer at pH 6.5, 150 mM NaCl. Fractions containing human DBNDD1 were pooled and concentrated. Sample purity was verified by SDS-PAGE and mass spectrometry. The final concentration of the human DBNDD1 NMR sample was about 300 µM.

Of note, the used construct has a thrombin cleavage site between the N-terminal His_6_ tag and the native human DBNDD1 sequence. Although no canonical thrombin cleavage site is predicted within human DBNDD1 sequence, the addition of thrombin led to the rapid protein degradation. Therefore, the removal of the purification tag was omitted, and the amino acid numbering is as follows: −19 to 0 indicates the purification tag, the native human DBNDD1 sequence starts with methionine number 1.

### NMR spectroscopy

^1^H-detected NMR spectra on human DBNDD1 were recorded at 283.2 K on a 700.5 MHz Bruker AvanceIII NMR spectrometer system equipped with a 5 mm TXI triple resonance probe (Bruker Biospin GmbH, Rheinstetten, Germany). Spectra with direct ^13^C detection were recorded at 293.2 K on a Bruker AvanceIII 700 MHz spectrometer equipped with cryogenic TXO probe at CERM/CIRMMP (Florence, Italy). The spectrometers were locked on D_2_O.

For direct ^1^H chemical shift referencing as 0.00 ppm we added 3-(trimethylsilyl)propane-1-sulfonate (DSS) at a final concentration of 0.1 mM to the NMR samples. ^13^C and ^15^N chemical shifts were referenced indirectly to the ^1^H DSS standard by the magnetogyric ratio (Wishart et al. 1995).

We assigned the backbone and side chain chemical shift resonances from a set of two- and three-dimensional ^1^H-detected heteronuclear experiments: [^1^H,^15^N]-HSQC, aliphatic and aromatic constant-time [^1^H,^13^C]-HSQC, HNCO (Ikura et al. 1990; Kay et al. 1990), HN(CA)CO (Clubb et al. 1992), HNCA (Kay et al. 1990; Grzesiek and Bax 1992a; Farmer et al. 1992), HN(CO)CA (Bax and Ikura 1991; Grzesiek and Bax 1992a), HNCACB (Grzesiek and Bax 1992b; Wittekind and Mueller 1993), HN(CO)CACB (Grzesiek and Bax 1992c), CC(CO)NH (Grzesiek et al. 1993) and [^1^H,^15^N]-TOCSY-HSQC (Marion et al. 1989). The sequential assignment, mainly of the proline residues, was accompanied by a series of additional 2D and 3D ^13^C-detected experiments using CON, (H)CACO, (H)CBCACON, and (H)CBCANCO, respectively (Bermel et al. 2009; Pontoriero et al. 2020).

The three-dimensional ^1^H-detected experiments were recorded with 25% non-uniform sampling. Compressed sensing with an iteratively reweighted least squares algorithm was used for data reconstruction (Kazimierczuk and Orekhov 2011; Holland et al. 2011). All spectra were processed using Bruker Topspin 3.6.2 or 4.1.1 and analyzed using CcpNmr Analysis 2.5 (Vranken et al. 2005) within the NMRbox virtual environment (Maciejewski et al. 2017).

### Structure prediction

For the sequence-based prediction of structural disorder we used the ODiNPred web server (https://st-protein.chem.au. dk/odinpred) (Nielsen and Mulder 2019; Dass et al. 2020). Figure 5A(I-II) shows the ODiNPred disorder prediction of human DBNDD1. ODiNPred predicts fully disorder approximately for the first 50 amino acids (residues M1-A49) in the N-terminal part, followed by a stretch of roughly 50 amino acids where the fractional formation of local order is predicted. After a short stretch (residues E98-R113) of fully disorder the partial formation of local order is also predicted for the C-terminal part (residues E140-D158) of DBNDD1.

According to the predicted structural disorder, we used the POTENCI tool (https://st-protein02.chem.au.dk/potenci) to calculate the random coil chemical shifts for human DBNDD1 based on the amino acid sequence considering temperature, pH value and ionic strength (Nielsen and Mulder 2018).

Additionally, we used the programs SSP (Marsh et al. 2006) and TALOS-N (Shen and Bax 2013), respectively, to examine potential secondary structure elements of DBNDD1 based on the assigned backbone chemical shifts.

### Extent of assignments and data deposition

By using a set of two- and three-dimensional NMR experiments (s. Methods and experiments) we achieved the sequence specific resonance assignments for nearly all backbone ^1^H, ^13^C and ^15^N spins of human DBNDD1. We could assign 99% of the backbone resonances (C^*α*^, C′, N′, H^*N*^). For the side chain protons and carbons (ß, γ, δ, and ε positions) the assignment could be completed to 73% and 76%, respectively. Table 1 summarizes the extent of assignment.

**Table 1:** Extent of backbone and side chain assignment of human DBNDD1

Nucleus	Assigned (%)	Total number
^1^H^*N*^	100	142 out of 142^a^
^15^N′	100	158 out of 158
^13^C′	100	158 out of 158
^1^H^*α*^	90	154 out of 171
^1^H^*β*^	87	218 out of 252
^1^H^*γ*^	79	137 out of 173
^1^H^*δ*^	51	53 out of 103
^1^H^*ε*^	24	10 out of 42
^13^C^*α*^	99	157 out of 158
^13^C^*β*^	99	144 out of 145
^13^C^*γ*^	70	96 out of 137
^13^C^*δ*^	54	55 out of 101
^13^C^*ε*^	50	7 out of 14

^a^16 out of the 158 residues in human DBNDD1 are prolines

**Fig. 2:**
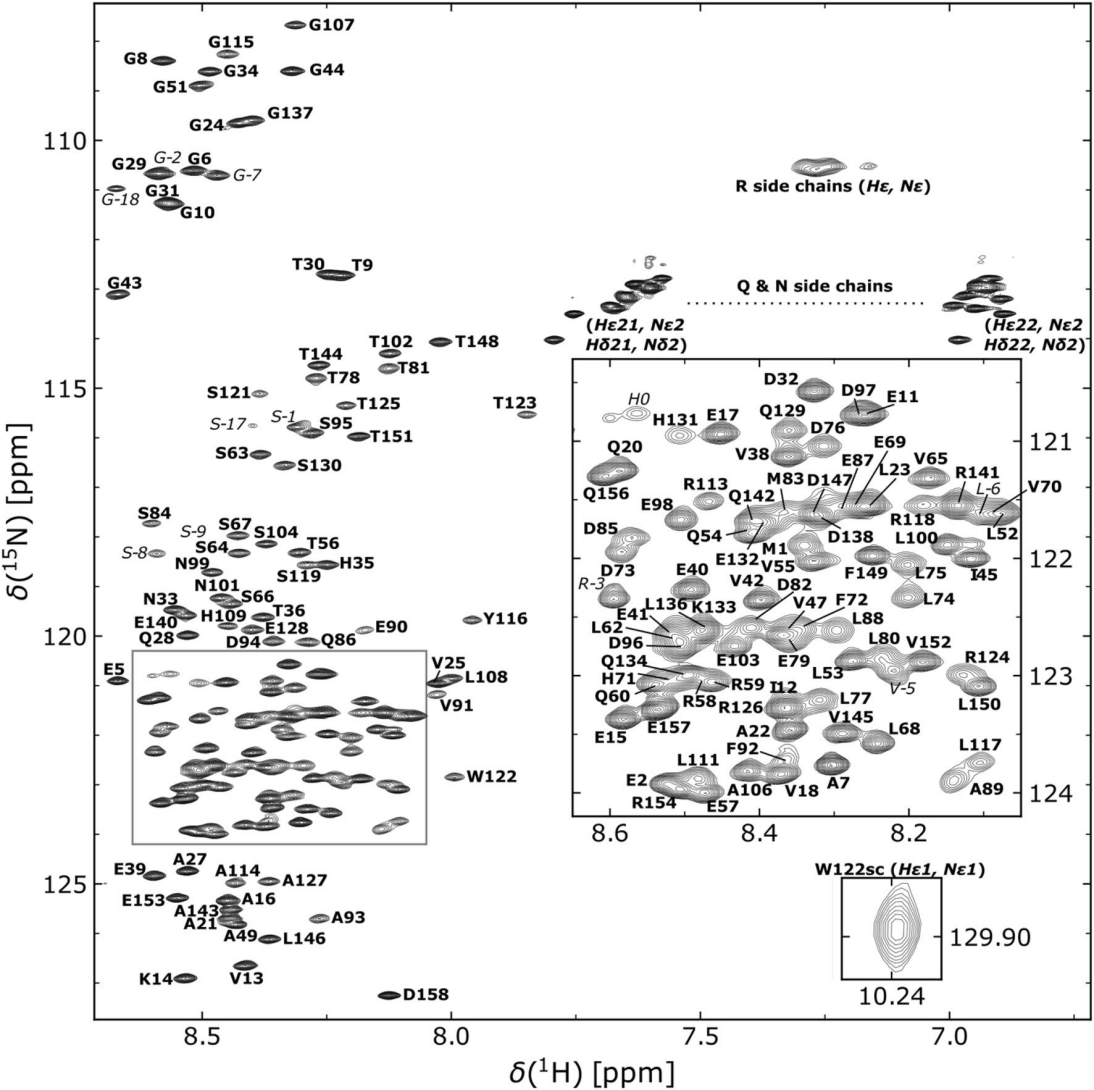
[^1^H,^15^N]-HSQC spectrum of ^13^C,^15^N-labeled human DBNDD1 in 10 mM NaPi, pH 6.5, 150 mM NaCl, 0.1 mM DSS, 90% H_2_O/10% D_2_O at 283.2 K, recorded at 700.5 MHz. Assigned residues are annotated in bold face one letter amino acid code according to the human DBNDD1 protein sequences (UniProtKB: Q9H9R9). Residues originating from the N-terminal purification tag are marked in italic. Non-degenerate protons of the side chain amino groups are connected by a dashed line

In agreement with a predicted low overall secondary structure content, the [^1^H,^15^N]-HSQC spectrum of human DBNDD1 shows limited signal dispersion in the ^1^H^*N*^ dimension (Fig. 2).

**Fig. 3:**
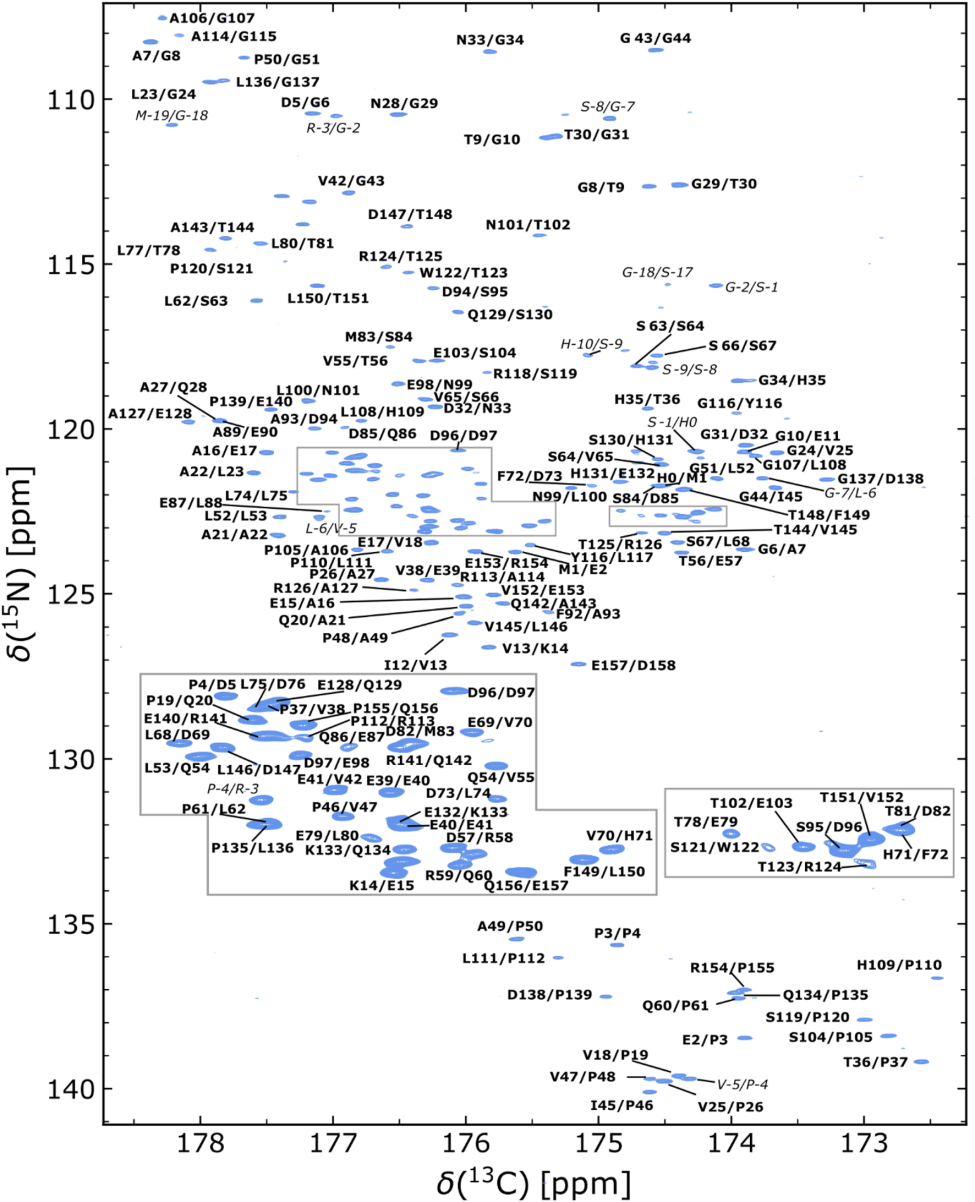
[^13^CO,^15^N]-spectrum of ^13^C,^15^N-labeled human DBNDD1 at 293.2 K. Assignments for backbone ^13^CO,^15^N correlations of neighboring residues are annotated in bold face. Assignable resonances originating from the N-terminal purification tag are marked in italic

The backbone ^13^CO, ^15^N-correlations of neighboring residues in the 2D CON experiment are given in Fig. 3.

**Fig. 4:**
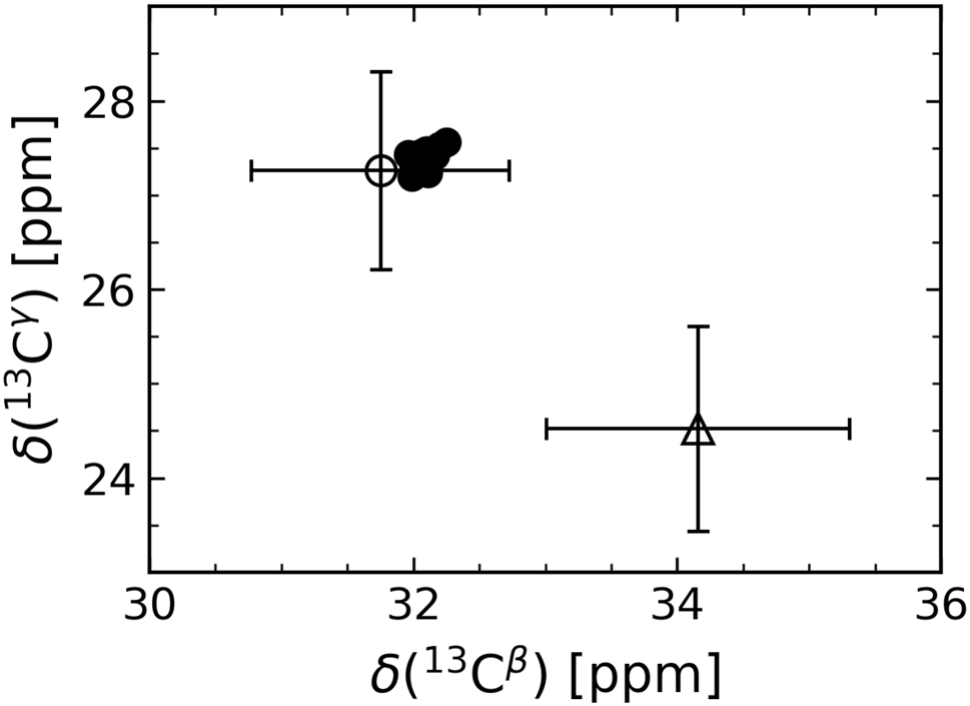
Proline ^13^C^*β*^ and ^13^C^*γ*^ chemical shift analysis for human DBNDD1 reveals all Xaa-Pro peptide bonds in *trans* conformation. Filled circles correspond to the assigned proline ^13^C^*β*^ and ^13^C^*γ*^ chemical shifts. 15 out of 16 prolines were completely assigned (C^*γ*^ of P120 is unassigned). The open circle and the open triangle indicate the location of the mean (standard deviation shown as error bars) for a proline in *trans* and *cis* conformation, respectively (Schubert et al. 2002; Shen and Bax 2010)

We assigned the ^13^C^β^ and ^13^C^γ^ resonances for 15 out of the 16 proline residues in DBNDD1. The ^13^C^γ^ resonance assignment of proline residue P120 is missing due to signal ambiguity. All assigned proline residues show ^13^C^β^ and ^13^C^γ^ values in the range of 32.09 ± 0.08 ppm and 27.42 ± 0.09 ppm, respectively, with a mean difference of the proline ^13^C^β^ and ^13^C^γ^ chemical shifts of 4.68 ± 0.08 ppm. The obtained proline ^13^C^β^ chemical shift values are plotted versus the ^13^C^γ^ chemical shift values in Fig. 4. Based on the ^13^C^β^ and ^13^C^γ^ chemical shift values, we assume that in its major conformation all completely assigned proline residues of human DBNDD1 are in a *trans* configuration (Schubert et al. 2002; Shen and Bax 2010). Moreover, the absence of an additional subset of peaks with lower intensity in the proline specific region of the CON spectrum (Fig. 3) supports the statement that all prolines are exclusively in *trans* configuration.

**Fig. 5:**
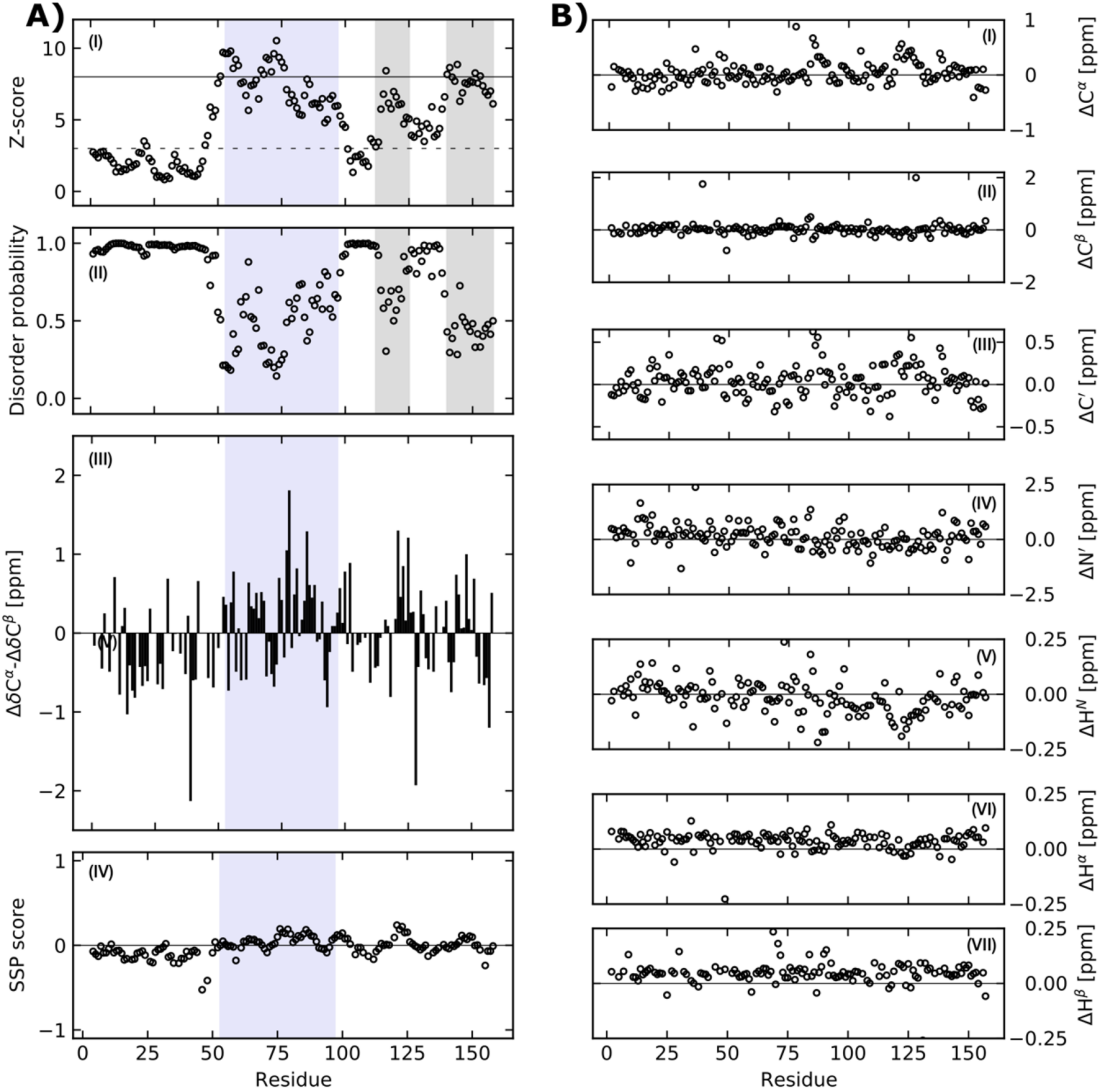
**A** The sequence-based ODiNPred analysis (A), I-II) of human DBNDD1 predicts the fractional formation of local order for the proposed dysbindin domain (residues L53-D97 are shaded in light blue). Additionally, two regions with low predicted disorder propensities are in the C-terminal part of DBNDD1 (shaded in light grey). The N-terminal part is predicted to be fully disordered. The circles show the residue-specific Z-score (A), I) and disorder probability (A), II). A residue specific Z-score larger than 8 (solid line) indicates structural order while a Z-scores below 3 (dashed line) predicts fully disorder. Z-scores between 3 and 8 reflect transient local structure propensity. The Z-score and disorder probability were calculated using the ODiNPred webserver (Dass et al. 2020). The differences between secondary chemical shifts of ^13^C^*α*^ and ^13^C^*β*^ resonances (A), III) and the secondary structure propensity (SSP) prediction (A), IV) based on chemical shifts were calculated using the SSP script (Marsh et al. 2006). A positive and negative SSP score reflect α-helix and β-sheet propensities, respectively. A SSP value of 1 reflects fully formed helical-structure and a value of -1 fully formed β -structure, respectively. Only ^13^C^*α*^, ^13^C^*β*^ and ^1^H^*α*^ chemical shifts of non-proline preceding residues were applied when running the SSP script. **B** Secondary chemical shifts analysis reveals that the human DBNDD1 is highly disordered throughout the entire protein sequence. Chemical shift differences calculated from the experimentally determined and predicted C^*α*^, C^*β*^, C′, N′, H^*N*^, H^*α*^, H^*β*^ chemical shifts (B), I-VII). POTENCI (Nielsen and Mulder 2018) was used for sequence-based chemical shift prediction

We used the obtained chemical shifts of human DBNDD1 for an initial structural analysis based secondary chemical shifts. The differences between the secondary ^13^C^*α*^ and ^13^C^*β*^ chemical shifts and the secondary structure propensity (SSP), respectively, (Fig. 5A), III-IV) were calculated using the SSP script (Marsh et al. 2006). An overall intrinsic disorder of DBNDD1 is supported by the application of secondary chemical shifts and the sequence specific SSP method. Although consecutive positive and negative differences of secondary ^13^C^*α*^ and ^13^C^*β*^ chemical shifts are observable, their magnitude are comparatively low to predict reliably secondary structure elements. The SSP method combines C^*α*^, C^*β*^ and H^*α*^ chemical shift values into single residue specific scores. The calculated SSP scores predict the entire human DBNDD1 protein as highly disordered (Fig. 5A, IV). In contrast to the sequence-based disorder prediction, an analysis based on the measured chemical shifts also reveals the proposed dysbindin domain (residues L53-D97) as highly disordered. The mean SSP score is − 0.016 ± 0.113 and by averaging the calculated SSP scores, an overall total of only 3.2% α-helical and 5.5% β-sheet structure is estimated for human DBNDD1. Additionally, we compared the experimentally determined chemical shifts with random coil chemical shifts, predicted at our experimental conditions using the POTENCI web server (Nielsen and Mulder 2018). The measured and predicted C^*α*^, C^*β*^, C′, N′, H^*N*^, H^*α*^ and, H^*β*^ chemical shift values agree remarkably (Fig. 5B, I-VII). The mean differences between the experimental and POTENCI-predicted random coil chemical shift values for human DBNDD1 are ΔC^*α*^ = 0.04 ± 0.19 ppm, ΔC^*β*^ = 0.03 ± 0.27 ppm, ΔC′ = 0.04 ± 0.18 ppm, ΔN′ = 0.14 ± 0.53 ppm, ΔH^*N*^ = -0.02 ± 0.08 ppm, ΔH^*α*^ = 0.04 ± 0.04 ppm, and ΔH^*β*^ = 0.05 ± 0.05 ppm.

Together, our experimental data and the secondary structure prediction based on them clearly show that human DBNDD1 is an IDP under buffer conditions chosen to somewhat mimic cellular conditions while providing optimal conditions for NMR spectroscopy. However, it is still speculative if the proposed dysbindin domain or parts of the C-terminal region prone for fractional local order are molecular recognition features that might fold upon binding. In addition, the effect of potential post-translational modifications on the structural dynamics of DBNDD1 remains elusive. It is likely that in a cellular context certain serines, threonines or the tyrosine are phosphorylation sites.

The inherent flexibility of IDPs renders NMR spectroscopy a suitable method to study the presence of local conformational preferences at a molecular level. Here, we report the backbone and side chain NMR resonance chemical shift assignments and provide an initial chemical-shift-based secondary structure analysis of the hitherto structurally “unknown” human protein DBNDD1. Hopefully, we can lay a foundation to adequately describe the fluctuating conformational behavior of DBNDD1 at atomic resolution and, thereby to gain a better understanding of DBNDD1 function and regulation in a cellular context.

## Data Availability

The assigned ^1^H, ^13^C and ^15^N chemical shift values of the human DBNDD1 are available in the BMRB (https://bmrb.io) under the Accession No 51301.
